# Generalized Dystonia in a Patient With Wilson Disease 5 Years After Liver Transplant: A Case Report

**DOI:** 10.5334/tohm.1120

**Published:** 2026-02-13

**Authors:** Elise Edwards, Benjamin Coleman, Matthew Feldman, Jude Hassan Charles, Danielle S. Shpiner

**Affiliations:** 1 Department of Neurology, University of Miami Leonard M. Miller School of Medicine, Miami, FL 33136, US

**Keywords:** Wilson disease, liver transplant, dystonia, tremor, case report

## Abstract

**Background:**

Liver transplant (LT) is considered curative for Wilson disease (WD) with hepatic failure refractory to medical therapy, particularly when neurologic symptoms are absent.

**Case Report:**

A 29-year-old man with WD developed progressive generalized dystonia five years after LT. He presented with acute-on-chronic neck pain, dysphagia, and dystonic posturing of the neck, trunk, and upper and lower extremities. MRI brain and copper studies were normal. Genetic testing confirmed two heterozygous pathogenic *ATP7B* variants. Symptoms improved with botulinum toxin injections.

**Discussion:**

Post-LT neurologic complications may arise from copper dysregulation, immunosuppressant neurotoxicity, or unrelated primary dystonia. Early recognition enables effective symptomatic management.

## Introduction

Wilson Disease (WD) is a rare autosomal recessive disorder caused by mutations in *ATP7B*, which encodes copper-transporting ATPase 2, primarily expressed in the liver [1]. Dysfunction of the protein leads to problematic copper accumulation predominantly in the liver, eyes, and brain due to impaired copper excretion and decreased synthesis or rapid degradation of ceruloplasmin, or both [1]. In addition to the risk of liver failure, copper accumulation can cause debilitating neurologic symptoms such as tremors, rigidity, dysarthria, personality changes, anxiety, and hallucinations [1]. Medical management includes copper chelation, oral zinc, and a low-copper diet [1]. Gene therapy has also proven effective [2]. When these fail, liver transplant (LT) is considered to be curative, especially in patients without prior neurologic symptoms [1]. However, neurologic symptoms have been reported post-LT due to suboptimal transplant copper excretion [3], low-copper diet nonadherence [3], and transplant surgery-associated copper release from the liver into circulation [4]. We describe an unusual case in which a WD patient developed new-onset dystonia 5 years after LT.

## Case Description

At age 13, the patient was diagnosed with WD via routine labs and biopsy confirmation. He was treated with intermittent trientene and zinc therapy before undergoing orthotopic LT with duct-to-duct biliary anastomosis for acute liver failure despite medical therapy (Table 1). Pre-transplant neurologic exam and evaluation by a pediatric neurologist were unremarkable, though he had a history of anxiety and autism spectrum disorder (ASD). Pre-transplant ophthalmologic examination was negative for Kayser-Fleisher (KF) rings. Pre-transplant MRI was unremarkable except for an incidental small left temporal fossa arachnoid cyst. Post-transplant, liver function improved and tacrolimus 4 mg PO BID (with early post-transplant trough goal of 8–10 ng/mL) was started to prevent rejection, and eventually tapered down to 1 mg PO BID. Trientene and zinc were discontinued post-transplant, but a low-copper diet was encouraged.

**Table 1:** Timeline of events in patient with Wilson disease.


AGE	EVENT

19	Underwent LT due to acute liver failure in WD despite treatment with trientine and zinc No reported neurologic symptoms Improvement of liver function Started on tacrolimus

24	Onset of chronic neck pain with tightness, burning, and allodynia Pain radiating to fingertips, worsened by movement Mild relief with ibuprofen, chiropractic adjustments, and botulinum toxin

29	ED visit for worsening neck pain, dystonic tremors noted Workup: Normal CT, MRI, and copper studies; tacrolimus slightly low Genetic testing confirmed Wilson’s disease (*ATP7B* mutations) Treatment: Cyclobenzaprine, physical therapy, onabotulinum toxin → improvement


Abbreviations: LT = liver transplant; WD = Wilson Disease; ED = emergency department.

At age 29, he presented with acute-on-chronic neck pain, describing 5 years of constant tightness, burning, and allodynia at the skull base and upper neck, radiating to the fingertips bilaterally. His pain was alleviated by lying down or supporting his head with his hands, and worsened with talking, chewing, swallowing, and typing. He also noted his head and upper extremity movements were less smooth. Prior workup included a reportedly normal MRI brain and laryngoscopy. Ibuprofen, chiropractic adjustment, and one round of botulinum toxin injections to the temporomandibular joint previously provided mild relief. At presentation, he was taking amlodipine 10 mg PO daily for primary hypertension, cyclobenzaprine 5 mg PO nightly for muscle spasm, escitalopram 10 mg PO daily for depression and anxiety, and tacrolimus 1 mg PO BID for post-LT immunosuppression.

Neurological examination revealed dystonic tremors and posturing of the bilateral upper extremities and neck, worsened with writing and head rotation. He had limited neck rotation, rightward tilt, and a “no-no” tremor when turning right. Truncal jerks transmitted to the head and neck were also present. Writing with the right hand induced a jerky tremor of the right arm, while writing with the left induced flexion of the first two digits of his right hand. Writing also induced plantarflexion and inversion of the left foot (Video 1). Brain MRI, including diffusion-weighted images, did not reveal any acute or suspicious intracranial abnormality, though the focal enlargement of CSF space along the anterior medial aspect of the left temporal fossa seen on his pre-transplant MRI was visualized again (likely representing an incidental small arachnoid cyst). There was notably absence of T1 hyperintensity of the globus pallidus internus. Cervical MRI demonstrated a small disc osteophyte complex indenting the ventral thecal sac without spinal canal or foraminal stenosis in the C3-C7 disc space. Neck CT was unremarkable. Serum copper (76 mcg/dL; normal lab range 70–175 mcg/dL), 24-hour urine copper (24 mcg; normal lab range 15–60 mcg), ceruloplasmin (19 mg/dL; normal lab range 15–30 mg/dL) and tacrolimus levels (2.7 ng/mL; normal lab range 5–20 ng/mL) were normal. Genetic testing via the Invitae Dystonia Panel revealed two heterozygous pathogenic *ATP7B* variants [c.2123T>C (p.Leu708pro) and c.2428G>T (p.Glu810*)], consistent with WD. No other pathologic variants were identified.

**Video 1:** **Neurological Examination Demonstrating Dystonia and Associated Hyperkinetic Movements**. **0:00–0:20**: While performing finger tapping with the arms extended, dystonic tremors and posturing of the bilateral upper extremities and neck are present. Intermittent truncal jerks are transmitted to the head and neck. **0:20–1:18**: The patient exhibits limited neck rotation and tilting. With active head turning to the right, a mild “no-no” head tremor emerges, accompanied by abnormal neck posturing. Flexion and extension of the neck are full. **1:18–1:54**: When writing with his right hand, there is task-induced jerky tremor of the right upper extremity with superimposed irregular, brief myoclonic-appearing movements. Simultaneously, there is a slight extension of the left thumb, and involuntary plantarflexion and inversion of the left foot are observed. **1:54–2:27**: When writing with the left hand, there is a dystonic posturing of the right hand, including flexion of the first two digits, along with recurrence of left foot inversion and plantarflexion.

He continued cyclobenzaprine 5 mg PO nightly as needed, started physical therapy, and began receiving onabotulinum toxin injections (dilution: 5units/0.1cc: initial dose was 25 units to right levator scapulae, 25 units to bilateral sternocleidomastoid, 25 units to bilateral cervical and supraspinatus trapezius portions, and 20 units to right lumbricals), with ongoing regimen adjustments per symptoms with sustained benefit.

## Discussion

LT is considered curative in WD because the transplanted liver expresses functional *ATP7B* and restores physiologic copper excretion. Nevertheless, rare cases of *de novo* neurologic symptom development following LT have been reported [5,6], highlighting the complexity of neurologic involvement in WD even after definitive hepatic treatment. This case illustrates an uncommon but clinically important scenario in which progressive dystonia emerged five years after LT in a patient with no pre-transplant neurologic involvement, normal post-transplant copper indices, and unremarkable neuroimaging. The delayed timing, phenomenology of the movement disorder, and treatment response raise important questions regarding the underlying mechanisms of his neurologic symptoms.

Historically, LT has been viewed as more effective at preventing than reversing neurologic disease [7], leading to hesitancy of its use in patients with prominent neurologic symptoms. However, more recent evidence challenges this idea. A systematic literature review by Litwin et al. [8] demonstrated major improvement or complete resolution of neurologic symptoms in 71.2% of WD patients with pre-existing neurologic involvement who underwent LT, prompting reevaluation of LT as a therapeutic option even in severe neurologic cases. These findings underscore that LT is not only hepatically curative but frequently neurologically beneficial, making the delayed emergence of dystonia in our patient—who lacked pre-transplant neurologic involvement—particularly unexpected.

Several potential etiologies merit consideration. Among these, we believe this patient’s presentation is most consistent with a primary dystonia that coincided with WD, rather than being directly caused by it, with other mechanisms remaining possible but less likely.

The timing of symptom onset strongly argues against perioperative or acute post-transplant copper redistribution as the primary mechanism. Neurologic worsening attributed to copper release during LT has typically been reported within days to weeks of surgery [6]. In contrast, this patient developed symptoms approximately five years after transplantation, making a surgical copper release effect unlikely. Moreover, longitudinal biochemical evaluation demonstrated normal serum copper, 24-hour urine copper, and ceruloplasmin levels, with stable graft function. Although normal copper indices do not entirely exclude subtle dysregulation in post-LT WD patients, the absence of biochemical abnormalities argues against active Wilsonian copper toxicity at the time of symptom development.

The clinical phenomenology further supports a diagnosis of primary dystonia rather than recurrent Wilsonian neurologic disease. The patient demonstrated task-activated dystonic posturing involving the neck, upper extremities, and left lower extremity, with exacerbation during writing and sustained symptomatic benefit from targeted botulinum toxin injections. While the distribution extends beyond a strictly segmental pattern, this multifocal task-activated involvement is well described in primary dystonia syndromes and does not necessarily imply a progressive metabolic or neurodegenerative process [9]. In contrast, WD–related movement disorders more commonly present with mixed and evolving phenomenology, often including parkinsonism and dysarthria, and are frequently accompanied by radiographic abnormalities [10]. Absence of KF rings and normal MRI further support a non-Wilsonian etiology such as primary dystonia.

An alternative explanation is that extrahepatic *ATP7B* dysfunction confers a long-term susceptibility to neurologic disease. Although copper dysregulation in WD primarily results from impaired hepatic *ATP7B* function [11], the transporter is also expressed in the kidney, brain, heart, placenta, and lungs [12]. This raises the possibility that extrahepatic *ATP7B* dysfunction contributes to certain clinical features, such as progressive dystonia despite adequate therapy [13,14]. In a cohort studied by Mohr et al. [15], 4.4% of patients with a purely hepatic or asymptomatic presentation developed late (defined as 12 months after treatment initiation) neurologic worsening despite adequate copper management. The occurrence of neurologic progression in both medically treated and post-transplant patients suggests that factors beyond hepatic copper accumulation may contribute to disease evolution. In this framework, WD may lower the threshold for dystonia development without producing overt biochemical or structural abnormalities.

Neurotoxicity from chronic immunosuppressive drugs is another consideration. Calcineurin inhibitors (tacrolimus, cyclosporin A) have been implicated as a cause of neurotoxic effects including mental status change, seizures, dysarthria, myoclonus, and tremors, even at therapeutic concentrations [16,17,18,19]. Possible mechanisms include degeneration of calcineurin-containing neurons and mitochondrial dysfunction [19,20]. However, these are normally accompanied by characteristic white matter MRI changes [16]. In this patient, MRI was normal and tacrolimus levels were never above therapeutic range, making drug toxicity a possible but unlikely etiology.

Psychiatric symptoms are occasionally the first manifestation of neurologic changes in WD [1]. It is possible this patient’s prior anxiety and ASD represented early neurologic manifestations and his dystonia arose from progressive WD-associated structural changes not detected on MRI. In fact, dystonia-causing copper accumulation, microstructural white matter changes, and putaminal volumetric change can be missed on traditional MRI, pointing to exploration of alternative imaging modalities such as fluid-attenuated inversion recovery, diffusion-weighted imaging, susceptibility-weighted imaging, and arterial spin labeling [21]. While no such abnormalities were identified in this patient, subclinical structural changes cannot be fully excluded.

In conclusion, this case highlights that new-onset movement disorders after LT in WD should not be assumed to result from recurrent copper dysregulation. The delayed onset, normal copper studies, dystonia phenomenology, unremarkable neuroimaging, and robust response to botulinum toxin suggest that primary dystonia is the most likely explanation in this patient, with extrahepatic *ATP7B*-related vulnerability as a plausible contributing factor. Early recognition of dystonia and careful phenomenological assessment are critical, as effective symptomatic treatment can significantly improve quality of life even when the precise etiology remains uncertain.
